# Developing a Sexual Health Promotion Intervention With Young Men in Prisons: A Rights-Based Participatory Approach

**DOI:** 10.2196/11829

**Published:** 2019-04-29

**Authors:** Michelle Templeton, Carmel Kelly, Maria Lohan

**Affiliations:** 1 Queen's University Belfast School of Nursing and Midwifery Belfast United Kingdom

**Keywords:** sexual health, male, prison, health promotion, rights-based participation

## Abstract

**Background:**

The sexual health of young men in prisons is often among the poorest in any given country. They may have developed sexual behaviors that, from a public health perspective, are considered problematic and burdensome. These include poorer use of condoms and engaging in more frequent casual sex, resulting in higher rates of sexually transmitted infections, including HIV and viral hepatitis. Thus, young incarcerated men are a highly marginalized and socially excluded high-risk group, in greater need of sexual health education and services.

**Objective:**

The aim of this study was to create an innovative sexual health promotion intervention, made for and with young men in prisons, to encourage them to avail of regular sexual health checkups. This included developing a Web-based animated-style sexual health promotion intervention (1.42 min) coupled with upskilling the prison nurses to offer a partnership approach to prison health care. This paper focuses on the development of the intervention and the importance of the underpinning rights-based (RB) participatory intervention design.

**Methods:**

We employed an RB participatory approach and recruited 14 participants who attended 3 coproduction workshops held within a prison site in Northern Ireland, United Kingdom. A bespoke 3-day training for nurses beforehand, ensured they gained a deeper understanding of the determinants of poor sexual health. The coproduction team comprised young men, prison nurses, nurse sexual health consultant, media company representatives, and facilitator. Workshops focused on content, design, tone and medium of communication for a Web-based intervention that would be appealing and engaging for young incarcerated men.

**Results:**

A 1.42-min animation *Dick loves Doot* was created to promote a positive attitude toward sexual health checkups. The RB approach enabled the young men to participate, have their voices heard and see their stories reflected through the animation. The nurses’ capacities to protect, fulfill, and respect the young men’s rights to appropriate sexual health services and education was also enhanced. Evaluations confirmed that we successfully provided accurate sexual health information in a way that was engaging and accessible and that encouraged the young men to avail of the new prison sexual health services that were set up in the prison and now provided by nurses.

**Conclusions:**

The RB participatory approach to health advanced in this study provided a means to (1) gain invaluable insider knowledge to understand the impact of structural determinants on health and health inequalities and strategies by which to target young incarcerated men (2) create inclusive opportunities for developing bespoke targeted interventions, and (3) galvanize collaborative partnerships to disrupt the structures and processes that lead to and encourage health inequities. To reduce future risk, effective treatment, coupled with coproduced interventions that transmit relevant health messages in a relevant and meaningful way, is key to success.

## Introduction

### Background

Globally, the health and well-being of young men who enter prison are often among the poorest in any given country [1,2]. Moreover, men involved in the criminal justice system have some of the poorest sexual health with especially high rates of sexually transmitted infections (STIs), including HIV and viral hepatitis [3-9]. Most of these young men will have experienced circumstances of structural and political adversity and many will have been exposed to the *toxic trio* in relation to their risk of harm, that is, parental mental ill health, substance misuse, and domestic violence [10-13]. Due to their circumstances, lower socioeconomic status, and lower levels of education, they may have experienced chaotic lifestyles and developed risky sexual behaviors that are grounded in a particular form of hegemonic masculinity, which exhibits patriarchal attitudes toward women, sex, and contraception [14-16]. Such behaviors may include poorer use of condoms and engaging in more frequent casual sex with partners whose sexual behaviors also place themselves and others at increased risk of STI and HIV, etc, (eg, commercial sex workers and intravenous drug users) both during incarceration as well as before and after. In many ways, the prison population is reflective of marginalized disadvantaged men more broadly, but incarceration may further compound these young men’s marginalization, future economic prospects, as well as their potential to form safe and secure relationships [5,17]. Importantly, sexual health issues greatly affect not only economically and socially marginalized young men’s lives but also that of their partners and communities [15,18-21]. The public health issues in prison are one part of the risk and the public health issue once they leave is another [2,5,22]. Hence, to address sex-related and other communicable diseases effectively, prisoners are identified in national and international sexual health strategies and guidelines as a key population in greater need of targeted sexual health promotion, education, and services [2,23-30].

However, the challenge is how to engage and communicate about sexual health with marginalized incarcerated young men in a way that is informative and effective, while not reifying hegemonic representations of masculinity that underpin “ *high risk-low concern* behaviors, and in a way that respects, protects, and fulfills their human rights [18,19,29,31]. Equally important is the representation of partners who for the most part will be external to prison. To be meaningful, sexual health promotion must address partnering-up scenarios relevant to the young men’s lived experience both within and beyond prison walls. Men’s sexual behaviors and practices do not occur in a vacuum but intersect with the gender and power, emotions, wants, needs, and rights of another person. Therefore, there is also a need for a more gender-relational approach [21] to sexual health promotion that seeks to empower marginalized young men to engage better with their partners to look after each other’s sexual health together.

According to recent reviews [32-36], engaging men and boys in developing sexual health and well-being interventions is crucial to enable positive change for all. Tailored interventions that empower them by using male-friendly language, speaking to men’s potential, and ensuring young men’s active participation are effective [33,34,36-38]. The use of videos, dramas, and digital media has also been shown to be more successful for men than some cognitive behavioral change models [32,34,35,38-40]. In addition, providing men with opportunities to critique stereotypical gender ideologies and explore the sexual rights of women may encourage partner and peer support and communication, which are enablers to positive sexual health [28,29,37,40]. In relation to the prison environment, the creation of a nurturing and safe space within the prison setting is crucial for encouraging the relationship building between the facilitators and the young men; it is also critically crucial for the relationship building between the facilitators and the prison management [41]. Some research suggests that men in prison might have greater opportunities to engage with health services and in activities and therapies to improve their well-being [2,5,12,42]. The potential for them to regress in prison and develop poorer health behaviors, arguably greater access to drugs, is also a concern [43].

This paper describes the development of a pioneering Web-based sexual health promotion intervention made for and with young men in prisons from inception to production, alongside the development of a partnership approach to sexual health care between the primary care providers (prison nurses) and the young men they serve. We begin by describing the human rights-based (RB) approach employed, which resulted in a Web-based animated-style sexual health promotion intervention entitled *Dick loves Doot* (1.42 min). An RB approach was chosen as our preferred way of working with young incarcerated men, as in this approach, they are viewed as *rights-holders,* and mechanisms can be put in place to create an environment in which their voices can be heard, to enable them to participate in developing services that are relevant for them, as opposed to *duty-bearers* providing support or services on an assumed needs basis and the young men typically having no say in what action is taken.

Mechanisms for assisting *rights-holders* to claim their rights include the following:

- A *designated-listener*, who is aware of the situation of the rights-holders and duty-bearers, seeks to build the capacity of, and help, both.
- Framing the issues in relation to national and international law, legislation, and jurisprudence, by considering the accountability of duty-bearers and their obligations.
- Advocacy to duty-bearers on behalf of rights-holders with limited *voice*.
- Participation and empowerment of rights-holders to help themselves.

### Theoretical Approach

#### A Rights-Based Approach

There are many conceptual frameworks and guidance available for public health intervention development [44-50] that describe key implementation stages and their components, as critical links for the translation of sciences into public health services. However, we opted to employ an *RB* approach to accentuate the link among human rights (violations), health outcomes, and the development of contextually relevant and sustainable interventions. The human RB legal discourse was also a strong argument that helped to mobilize the state actors and get them on board to secure the inclusion, engagement, and participation of young men within the prison setting, and create an enabling environment whereby their voices could influence the end product [28,51]. For our project to conform to the standards of an RB project, we needed to fulfill the following 3 key principles:

1. The goal must further the realization of human rights.
2. The process must be guided by human rights standards and principles.
3. The outcome should strengthen the capacity of (1) state agents (duty-bearers) to meet their obligations, (2) rights-holders to claim their rights, via the processes of empowerment and accountability (United Nations, Statement of Common Understanding, 2003) [52].

There are a number of human rights standards relevant for prisoners that guide the process that are included in major international treaties [53,54]. These standards consistently emphasize that prisoners’ incarceration should not interfere with their human rights to health and education [2,23,26-29].

The ultimate goal in this project is to empower marginalized young men to further their right to health, which not only includes access to health services but also appropriate health-related education and information (information that is understandable and relevant to the target population). Applying human rights discourse in this study helps us to understand that poor sexual health outcomes for these young men are not simply issues of *public health* but are also the consequence of many structural and institutional rights violations, which marginalize them and their communities [51,53,55]. That is, they have *not* had adequate access to health services and *not* received appropriate education and information to enable them to make positive healthy decisions in relation to their sexual lives. Focusing on the young men’s right to good sexual health and related sexual health education and information rather than viewing their sexual practices as problematic highlights the inextricable link among individuals’ risk taking, human rights violations, and social and governmental responsibility and accountability, and this particular group’s poor sexual health outcomes [31].

The coproduction occurred alongside the development of a partnership approach to sexual health care between the primary care providers (prison nurses) and the young men they serve. The latter involved upskilling of the nurses to gain a deeper understanding of the lives and sexual health needs of incarcerated young men and develop the nurses’ ability to engage in more health promoting sexuality-related communication during both one-to-one and educational-style group consultations with their patients. The specific aim of the intervention then is to promote positive sexual health and sexual health checkups leading to a reduction of STIs. More broadly, our aim was to improve the health and well-being of marginalized groups of men by fostering a human rights and gender-relational focused approach, essentially to empower disadvantaged men to take responsibility for their own and their partner’s sexual health. In the remainder of the paper, we describe the development processes, and include the logic model, to demonstrate the decision making during the creation of the intervention, from design stage through to production and dissemination strategies.

**Figure 1:**
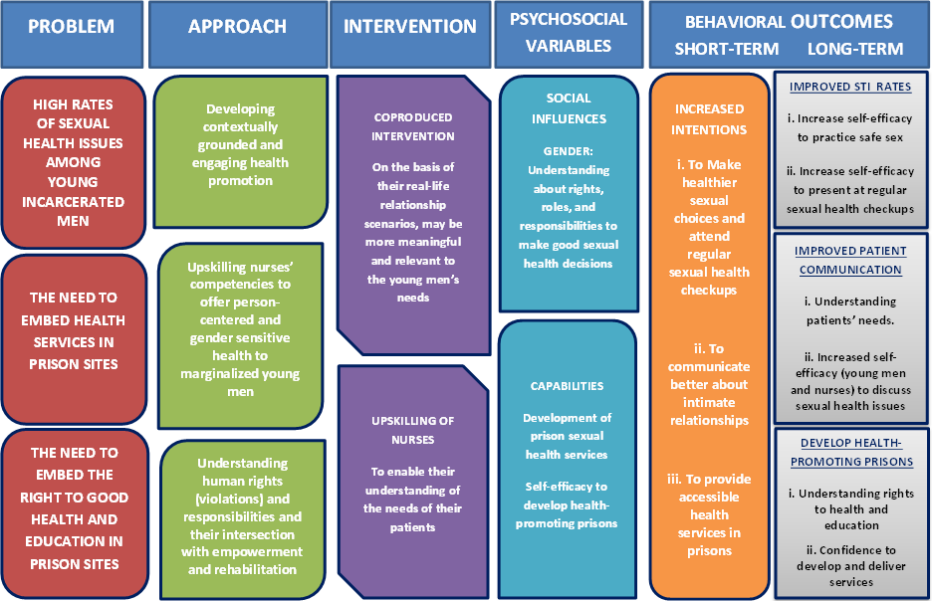
Project logic model.

It was this understanding that underpinned the logic model of the project (Figure 1), which demonstrates how we framed the problem, the ultimate goals, and the mechanisms to achieve these goals.

## Methods

### Research Design

Drawing from this RB axiom, outlined in Figure 1, the young men have the right to take part in educational activities to enhance their rehabilitation and their reintegration back into society. On the basis of the principles of inclusion, empowerment, capacity building, and accountability, the RB approach ensures that concepts such as respect, voice, and equality converge with participation rights [56-59]. In this way, the young men are viewed as experts on their situation and are invited to participate as consultants on the project to offer guidance and advice. The young men are identified as the rights-holders, whose rights to appropriate health and education may have been violated. The duty-bearers (agents of state—state employees) currently involved in the young men’s lives are (1) the prison nurses, who are obligated by law to uphold the young men’s rights by providing relevant sexual health information and treatment and (2) the prison staff, who are obligated to provide access to appropriate health and education for these young men.

We discussed our RB approach with the nursing and prison management staff who agreed to become involved in the project, and we also discussed that the young men had the right, the skills, aspirations, and expertise required to be included in the project also as consultants. This was important as for a participatory process to be successful and the end product to be relevant, we needed the right people around the table and all collaborators to share a mutual understanding of the expertise each brought to the team [60]. However, some ways of working may create distance with affected populations, which can impede the building of relationships on the basis of mutual respect [60,61]. Reflecting on the imbalances of power between the young men and prison officers, it was agreed that the prison officers, in this case, would be excluded from the working group. However, every effort was made to convey information about the study to keep them informed and invested, and their support was critical in allowing the young men to participate and providing the team with access, space, and technology to engage with the young men during our participatory meetings.

Consequently, the young men and the prison nurses were deemed the two significant parties to bring together in a collaborative partnership to create a useable solution. We sought to build their capacity around developing a mutual understanding of the determinants that cause poor sexual health outcomes and a lack of individual agency and autonomy for these young men, in the context of broader social structures [62,63]. In the collaborative partnership, key health messages that the young men need to know are shared by the health professionals and the young men share information on their experience of the issues and the reality of their lives. Both parties collaborate to decide how best to share positive sexual health messages with their peers in a way they will hear, listen to, and act upon. The coproduction approach to knowledge realizes the human rights of the young men by empowering them to share their relevant expertise, alongside *duty-bearers*, who are also empowered to fulfill their obligations to provide appropriate health-related education and information [61].

**Figure 2:**
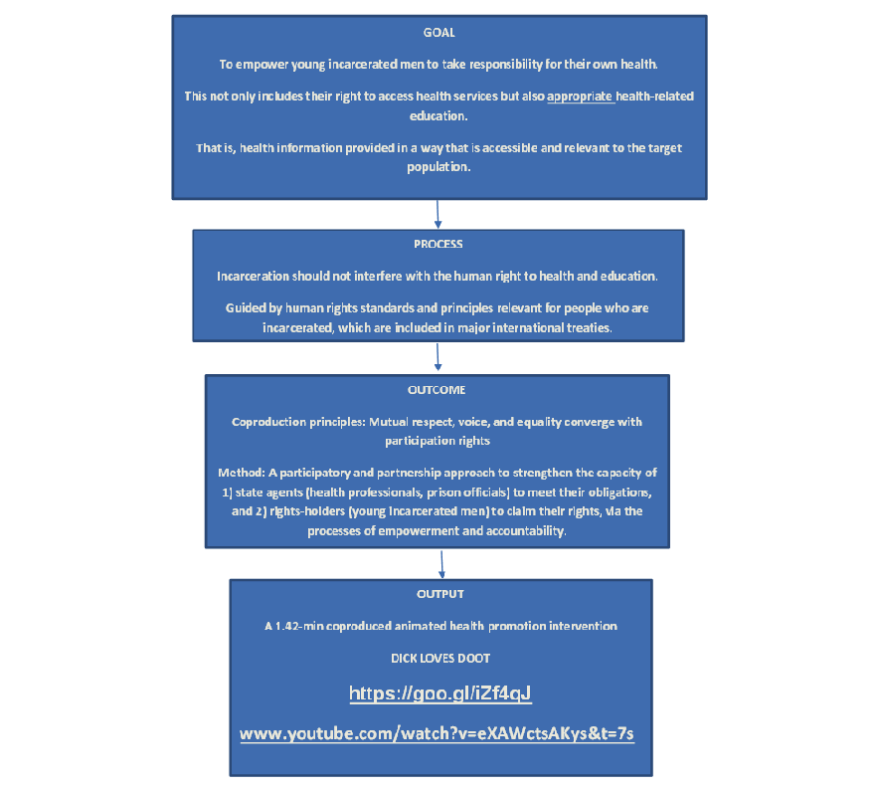
Overview of rights-based approach.

The products of this type of coproduction can lead to the creation of tailored and targeted interventions that are relevant and meaningful to the young men’s lives. This empowering approach can increase knowledge and awareness of gendered norms that intersect with intergenerational poverty and lack of social and economic opportunity for the young men. Developing health promotion messages together in this way can also increase both the nurses’ and young men’s sense of efficacy and control and it can create a greater sense of community and social support and thus ensure that the nurses protect, fulfill, respect, and deliver the young men’s rights to appropriate sexual health and education, which may lead to positive behavior change around sexual health communication and services, for both the health care professionals and their patients [62,63]. This paper focuses on the development of the intervention and the importance of the participatory intervention design with a preliminary evaluation of the young men’s participatory experience. Please see Figure 2 for an overview of the RB process.

### Research Setting

Hydebank Wood College (HBW) is one of 3 prison sites in Northern Ireland, United Kingdom. The college accommodates young men between the ages of 18 and 21 years and all female prisoners who are separately housed, 172 prisoners in total as of May 24, 2018. HBW college has a focus on education, learning, and employment, and it was the first young offenders’ institution in the United Kingdom to transition to *secure school* status (April 2015), which has required a major rethink about the role of punishment and the remit of prisons in young people’s lives. Notable changes have included the introduction of universal vocational training and employability skills and a new educational curriculum provided in-house, placed firmly at the heart of rehabilitation. Importantly, the inclusion of health behaviors, particularly relationships and sexuality education (RSE), is now being recognized as a critical element of the rehabilitation process within this prison context too [64]. The health promotion intervention is developed with the men’s prison section only. However, the development of the clinical sexual health service within the prison is made available to both the women and men.

Ethical approval was awarded by the Office for Research Ethics Committees Northern Ireland (reference: 17/NI/0082) and Governance permissions and security clearance from the Northern Ireland Prison Service and South Eastern Health and Social Care Trust. Verbal approval was given by the voiceover actors to use their image as part of the study.

### Participants and Procedures

There were 2 stages to the study, Stage 1 involved upskilling the prison nurses, and Stage 2 involved upskilling the young men by coproducing the sexual health promotion animation. The project ran over 1 year from January 2017 to December 2017.

#### Stage 1: Upskilling Nurses

##### Target Population

An important element in the RB approach is to build the capacity of all those identified to become involved to understand the issues of rights violations. The duty-bearer identified in this instance was the prison nursing team.

##### Sampling and Recruitment

Prison nurses (n=8) who work in the Northern Ireland prison service were selected because of their interest in further developing sexual health services within the prison context. The nurses received a 3-day in-depth training course, where they learned more about sexual health behaviors, education, and group facilitation skills with young men. This course was developed in collaboration with sexual health education specialists specifically for this project, and it had 2 strands. The first sought to improve the nurses’ knowledge and understanding of the young men’s lives and how the development of their sexual behaviors and practices may be linked to human rights violations in relation to their personal circumstances and lack of quality health and educational information. The second strand aimed at improving the nurses’ skills and personal competencies to deliver sexuality-related communication [29] and health promotion during sexual health consultations with young men in prisons. This could be one-to-one clinically-focused consultations, which was their typical way of working and also promoted their competency to move toward educational group style consultations within prisons.

Topics over the 3 days included up-to-date research on young people’s sexual behaviors and practices in general, and they included the results of recent research interviews conducted with the young men in their care, in relation to their understanding, wants, and needs around RSE. We also focused on sexual language and communication during sexual history taking, exploring personal sexual values and prejudices, legal issues, gender relations, sexual consent and exploitation, trauma and adverse childhood experiences, and the benefits of coproduction and participation. This helped the nurses develop a more psychosocial-oriented and deeper understanding of the needs of their patients’ and also helped to breakdown barriers between their personal values and their professional health provider role and address their levels of embarrassment and discomfort when discussing the expression of sexuality and sexual practices that they may not agree with or may find immoral. In an additional strand of the project, the nurses completed Web-based sexual health modules and received training and support in STI assessment, treatment, and management, to develop their competencies in these areas and also to set up sexual health clinics in the prisons. The clinical development strand of the project is reported elsewhere.

#### Stage 2: Coproduction Workshops With Young Men and Whole Group

##### Target Population

The participatory coproduction team comprised 14 members who met on 3 occasions during 2-hour workshops in HBW. The group comprised members of the research and nursing teams, and we required a sample of young incarcerated men to complete the group.

##### Sampling and Recruitment

The young men were recruited via the health care team within the prison site and chosen on the basis of their interest in the project and their ability to work together in a group situation. They were provided with information leaflets 1 week before a face-to-face meeting with the researcher who ran through all the information with them and reiterated what was expected of them. Their consent was then taken to become involved, and a meeting of the whole group was arranged for the following week. The group was constructed as follows:

- Young Men×6
- Consultant Nurse in Sexual Health×1 (CK)
- HBW Nurses×4
- Participation Facilitator×1 (MT)
- Media Company Representatives×2

##### Data Collection and Analysis

Data were collected during each workshop and the team would take this information away and report back their interpretation of this at the following workshop. The young men refined this interpretation as we went along checking that they were able to identify with the scenarios, language, and tone of the interpretation and see their lived experience reflected in the final scripts and images.

###### Workshop 1

After introductions, of all 14 members of the group as described above, workshop 1 focused on developing the purpose of the Web-based sexual health promotion intervention and developing the content for the intervention, that is, giving and receiving information among individuals to understand cultures, values, beliefs, and skills. Facilitated discussions centered on the following key issues around getting a sexual health check:

- Why is it important?
- How would you approach it?
- What can happen?
- What would you have to do?
- How can it be fixed?
- Partner notification

At times, the personality and behavior of the young men as they shared their experiences during our discussions made some members of the workshop, who may have less experience working with the young men, uncomfortable. It was the role of the facilitator to encourage the team to pay attention to the project’s central ideas and values and to listen to, understand, and reflect the concerns of the affected population. This realization helped bring everyone together around our common goal. Both parties exchanged views on the key issues and a picture emerged around the reality of the young men’s lives on the basis of their narrative that would shape the end product. The media company representatives took this information away to develop a creative storyline around the young men’s narratives and liaised with the research team to ensure the accuracy of the sexual health information.

###### Workshop 2

Workshop 2 focused on refining the storyline and discussing how to transmit our messages in an engaging medium of communication. An important consideration was the restrictions placed on the young men by being in prison and the technology to which they are denied access. For example, we ruled out mobile phone and digital apps as they would have no access to these technologies. Our preferred delivery method was to host our intervention on television screens throughout the prison site, such as visiting, and recreation areas. We decided to additionally host it on our research institution’s YouTube channel to freely share with other interested parties and allow greater accessibility to the general public. We focused on script and storyboard development, which was the backbone to the content of the intervention.

The media experts came to this workshop with a creative brief that comprised 2 scenarios. One was based on 2 characters meeting in a nightclub for the first time and the other was centered on a *sexpert* in a Physician’s office talking to a young couple about sexual health. They read both scripts aloud for the team and we all discussed the pros and cons of each. The young men decided that both scenarios were not overly relatable as nightclubs are not the only place they meet a sexual partner and they would never present at a *sexpert’s* office for relationships and sexual health advice with a new partner. Instead, they suggested keeping the scenario to a regular male and female character discussing between themselves about initiating a sexual relationship. This was very pleasing to the team who appreciated the importance of getting a sexual health check in the context of communicating with a new partner.

During this workshop, the young men told us that to be effective, an intervention for them would need to be pitched at the right language level including accent, it had to be realistic, easy to understand in terms of style and tone, and they added that the length should be *short and snappy*. The young men explained that a short video was the preferred medium to fulfill this brief. The media experts then showed numerous examples of this to the group on computer. After much deliberation, the young men decided on an animated explainer style, narrated video with eye-catching visuals. They reiterated that the tone of this was to be simple, short, snappy, and fun, for use on social and Web-based media, and they also commented that humor was essential.

###### Workshop 3

**Figure 3:**
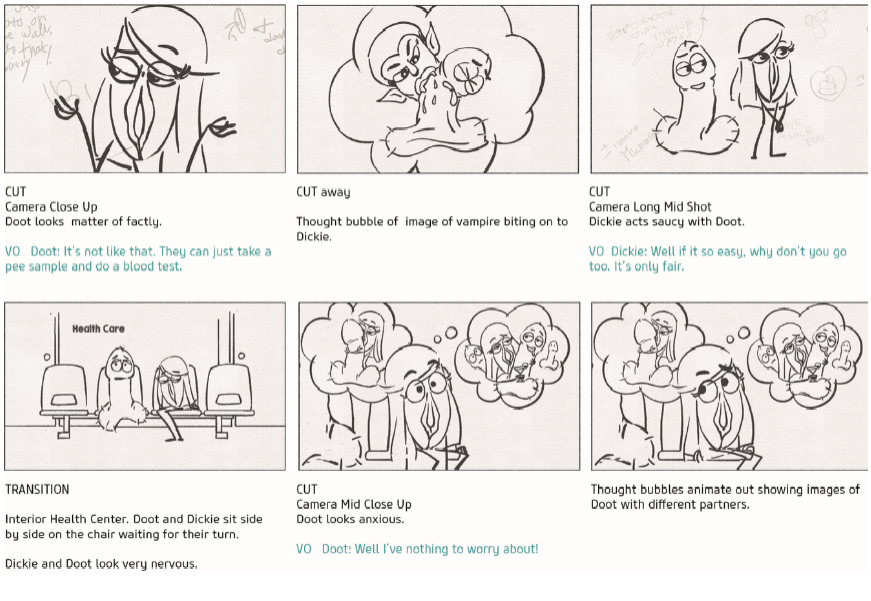
Example from the storyboard.

Workshop 3 focused on refining the language and visuals that would be truly representative of the young men’s lived experience. The media experts in collaboration with the research team developed a new script and storyboard (Figure 3) on the basis of the young men’s suggestion of 2 characters discussing their sexual health and negotiating having sex for the first time together. Again, we refined the script together on the basis of language the young men would actually use and agreed that humor was used effectively. We discussed the relatability of the characters and names were chosen *Dick and Doot*.

**Figure 4:**
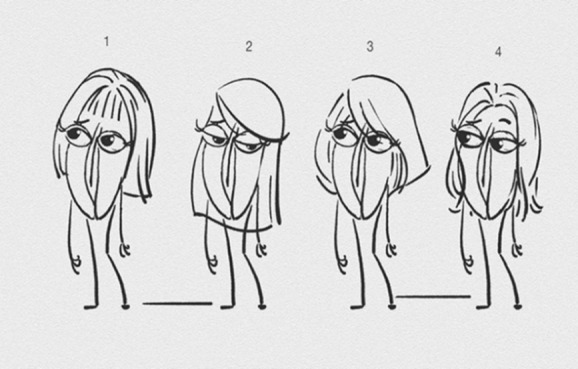
Refining the Female Character.

We also sought input from a group of 4 young women when refining the female character (Figure 4). The group unanimously agreed that caricature number 2 was representative of most young girls today, with long straight hair, eyelashes, and eyebrows. The team also agreed on the number and characteristics required of the voiceovers for the animation. A young male and a young female about 17 to 18 years of age with a Northern Ireland accent were deemed to be required to voice the characters of Dick and Doot. An adult female with a smooth, clear, upbeat voice was to be the third voice over. Participants prescribed that she would read an encouraging message at the end of the animation in an upbeat and positive way in an effort to normalize going for a regular sexual health checkup.

**Figure 5:**
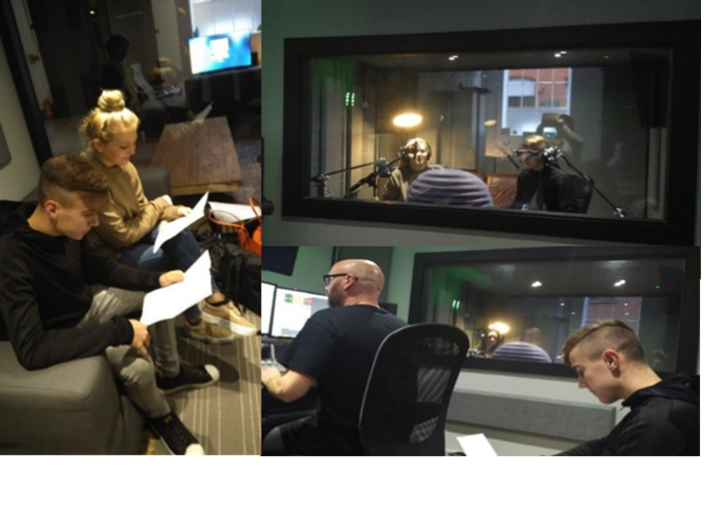
Voice over actors in the sound booth.

The media team hired 3 actors, a young male, a young female, and an older female, on the basis of the young men’s suggestions, to record the voiceovers to embed into the animation (Figure 5).

###### Final Sign-Off Workshop

**Figure 6:**
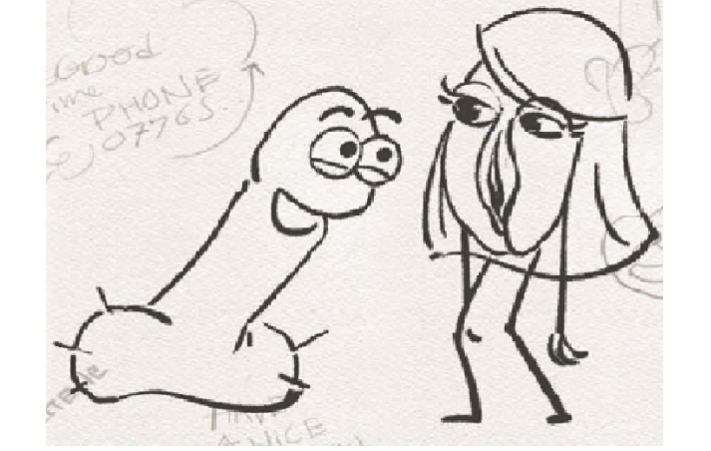
Screenshot from the intervention.

**Figure 7:**
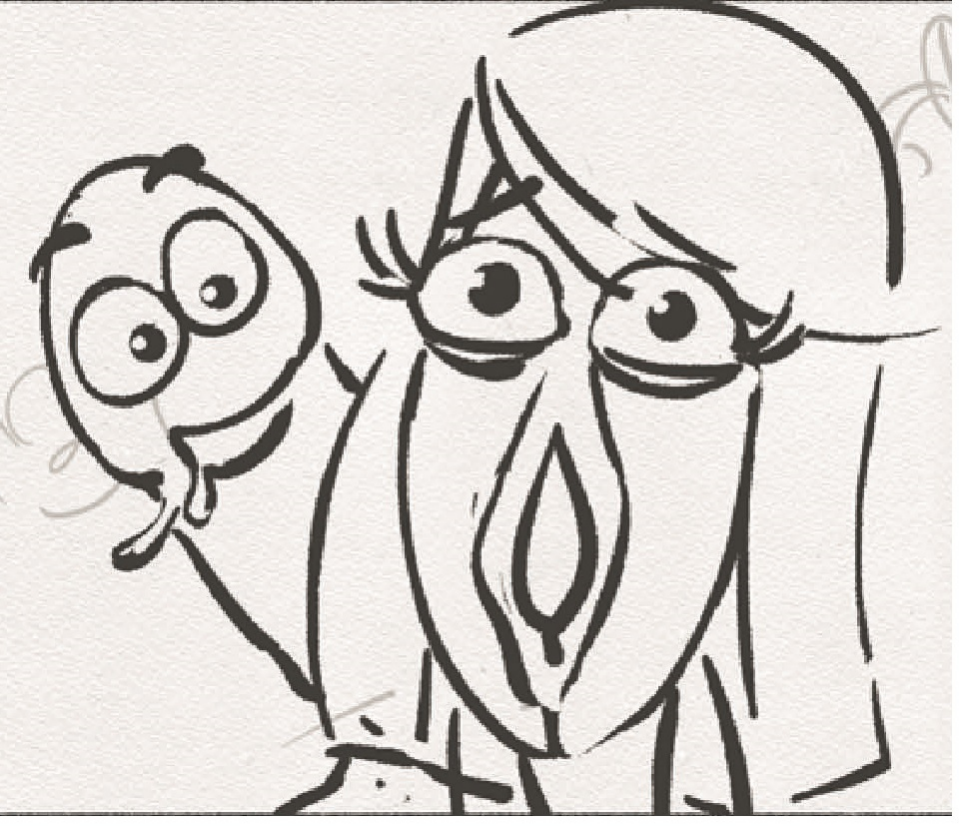
Screenshot from the intervention.

A final sign-off workshop was organized in Hydebank with the facilitator in which the penultimate storyboard was showcased to the young men who gave it a final seal of approval. Please see Figure 6 and Figure 7 for a screenshot from the intervention.

## Results

### Participation

In conversations during the workshops, the 6 young men in the group claimed they had gained a better understanding of the impact of their behaviors, and all self-referred to the nurses to have their first sexual health checkup. Having reviewed the final storyboard, they were excited for the launch of the animation, *Dick loves Doot*, which was launched at HBW on March 15, 2018. It is hosted on the Queen’s University Belfast, School of Nursing and Midwifery, YouTube channel [65]. (Please contact the authors if you are having trouble accessing the animation). It has been agreed to play this on television screens throughout all 3 Northern Ireland prisons, and we are also exploring useful contexts to use this with other young men via the university and with community health promotion colleagues. This promotion tool will also be included in the sexual health module of a broader program of work we are developing in the prison—a *Relationships and Future Fatherhood* intervention for young incarcerated men.

### Preliminary Evaluation

A focus group was arranged at the end of the project to explore issues around the experience of participation and the final product. Unfortunately, because of the transient nature of the prison population, only 2 members of the young men’s group were available to attend. Overall, they said they enjoyed their participation in the project, found it interesting, and fun:

everybody was all good, the work was all good, and everyone was 100%.

Male 1

oh aye we had good craic (fun) with them-ins aye, it was funny.

Male 2

whenever I was out there I went with someone but it did make me think…so I got checked anyway and tested when I came back in.

Male 2

They also regarded it as useful learning and made speaking up and asking questions about their bodies and their health easier:

its opened my eyes.

Male 1

it was great to get out (of their cell) and come over here and get a bit of craic, there’s certain questions that you’d be afraid to, reach out like, its mad, but it was easier to ask because you’s were already there.

Male 1

knowing that other people think about things the same way and have done similar things it’s easier to talk about what you’ve done cause sometimes you don’t wanna talk about things that you’ve done just in case you embarrass yourself.

Male 2

The young men agreed that their voices had been listened to and that the intervention reflected their real-life issues and concerns, while also portraying a clear message that would be useful to other young men.

it’s just spot on like everything was there.

Male 1

It’s gonna be good like its short and its funny as well, that’s the way you need it.

Male 2

They also felt grateful to, and valued by, the prison authorities for providing the opportunity for them to participate in something that was relevant to them and other young men:

fair play to them (prison officials) for letting you’s in to do this…the more you put in the more you get back.

Male 1

In relation to accessing the sexual health services that had been set up inside the prison, the young men had made full use of this, which is reflected in the number of tests being carried out in the clinics, and were encouraging others to do so as well:

before I went out I got checked and then whenever I came back in after going with her I got checked again, so I know.

Male 2

I got my bloods and urine and all done like, we all did.

Male 1

The nurses’ evaluations of the 3-day training course, which prepared them to better understand and engage with their patients on sexual health matters, described how much they enjoyed and greatly benefitted from this knowledge; they highly recommended the course to their colleagues who work in other prison sites:

The course was delivered by a great team who made it very interesting and funny at times. A lot was learned and I am glad that I got the chance to participate in this.

Nurse 1

I feel perhaps more nurses who work with patients doing sexual health checks on sites should go on this course. Currently I am the only nurse from my prison to attend this 3-day course.

Nurse 2

Importantly, the nurses described developing a completely fresh attitude on becoming upskilled to assess asymptomatic sexual health concerns, particularly in the manner in which they approached sexual health communication with their male patients. This also included, for the first time, delivering health promotion in a group setting with their patients within prison sites, and this experience exceeded their expectations. The following quote illustrates a common reaction from all the nurses on completing the course:

I have had a complete U-turn on previous view that 1-to-1 sexual health education was better than group work. Now a convert to the idea of group work!

Nurse 3

## Discussion

### Principal Findings

This study adds to the scant literature on conducting sexual health promotion research with hard-to-reach young men within prison and describes an empowering RB method on how to engage them in developing health interventions that are relevant to their particular needs, that is, *made by them, for them*. These young men tend to have multiple and complex problems and often lack the personal assets and opportunities on which to draw on to build healthy lifestyles [1,10,12]. This example describes how health care professionals can come together with a target audience to share information and communicate health messages in a way that is relevant and engaging [29].

This RB process used is designed to empower both the service-user (young men in prison) to take responsibility to improve their own health and *agents of state* (duty-bearers—nurses) to fulfill their obligations under human rights legislation to provide appropriate and accessible health and education [12]. Such an inclusive approach can reduce the young men’s risk behaviors upon release and assist with their reintegration back into the community [66]. Working within the prison environment and employing an RB approach provided an opportunity to work with a group of marginalized young men and support and advance the concept of *healthy prisons* [20,28,41], that is, to reinforce the idea that *prison health is public health* and that prisoners should benefit from rehabilitation strategies that empower them to be released back into the community in better health than they entered, for the good of themselves, their families, and communities [2,23,25,26,28].

This study also adds to our understanding of how health promotion can be cocreated in the prison context. According to our methodology, the responsibility is on *agents of state* to provide health and education information that is accurate, accessible, and useable. Our approach to developing fun and engaging sexual health promotion materials within prisons can help normalize sexual health among a high-risk population in an accurate yet accessible and meaningful way. Consequently, incarceration can provide an opportunity for health researchers to reach this high-need and typically hard-to-reach group. Although this situation can enable access to participants, an effort was still required on their part to continue to attend and engage at each meeting. Factors that fed into their engagement centered on the focus on their rights, and the amount of decision-making power and autonomy they were given to input into the content, design, and mode of communication of the Web-based intervention, which also added legitimacy to the end product [57,61]. The young men found the meetings fun and informative, which impelled them to attend. They learned lots, felt valued and listened to, and seeing their ideas reflected in the products created in them a sense of ownership and pride over the intervention.

Focusing on the needs of society’s most marginalized young men by helping them to maintain good sexual health is a powerful way of addressing health inequalities overall [15,16]. Situating their sexual health in the wider context of human rights is a strong argument for the inclusion of their voices, which have largely been missing in the sexual and reproductive health research and education agenda to date. The findings support the contention that a human RB approach to coproduction, particularly for marginalized groups, can add insider value to interventions that can empower incarcerated young men to make positive healthier decisions about their lives and their partner’s lives, and thus support their rehabilitation. The experience at Hydebank has shown that strong collaborations among academics, health and social care, prison management, and the young men themselves can be forged to support learning and skills development and enhance the overall rehabilitation experience of young men in prisons.

The support we received for our RB participatory work from the Northern Ireland Prison Service and the Department of Justice in Northern Ireland has been crucial to allow us to provide the space for prisoner voice to be heard, which was key to the success of this project. As such, Northern Ireland is leading the way in promoting innovative approaches to developing education and health behaviors as part of the rehabilitation of young men. This project demonstrates what can be achieved when agencies work together around a common goal of reforming and rehabilitating these young men to encourage good health behaviors within, and on leaving, their incarceration. Future steps of this research will be evaluation of the intervention.

### Limitations

Participants in this study were restricted to 1 prison site in Northern Ireland (United Kingdom), which could mean that the issues and priorities they presented may not be representative or generalizable. However, the literature suggests that their experiences are consistent with young men residing in other marginalized areas and contexts. The evaluation of the animation and participatory process discussed during the 1 focus group at the end of the project included only 2 participants who shared similar positive views. However, the broader generalization we advance in this study is the approach and not the product.

### Conclusions

The RB participatory approach to prison health advanced in this study can provide invaluable insider knowledge and strategies by which to target the health inequities that affect young incarcerated men. It provides a means to (1) understand the impact of structural determinants on health and health inequalities, (2) create inclusive opportunities for developing bespoke targeted interventions, and (3) galvanize collaborative teams to work together to disrupt the structures and processes that lead to, and encourage, health inequities. To reduce future risk, effective treatment, coupled with coproduced interventions that transmit relevant health messages in a way that is meaningful, is key to success.

## Abbreviations

HBW: Hydebank Wood College
RB: rights-based
RSE: relationships and sexuality education
STI: sexually transmitted infection
